# Novel Water Probe for High-Frequency Focused Transducer Applied to Scanning Acoustic Microscopy System: Simulation and Experimental Investigation

**DOI:** 10.3390/s24165179

**Published:** 2024-08-10

**Authors:** Van Hiep Pham, Le Hai Tran, Jaeyeop Choi, Hoanh-Son Truong, Tan Hung Vo, Dinh Dat Vu, Sumin Park, Junghwan Oh

**Affiliations:** 1 Faculty of Mechanical Engineering and Mechatronics, PHENIKAA University, Yen Nghia, Ha Dong, Hanoi 12116, Vietnam; 2 PHENIKAA Research and Technology Institute (PRATI), A&A Green Phoenix Group JSC, No.167 Hoang Ngan, Trung Hoa, Cau Giay, Hanoi 11313, Vietnam; 3 Industry 4.0 Convergence Bionics Engineering, Pukyong National University, Busan 48513, Republic of Korea; tranlehai999@pukyong.ac.kr (L.H.T.); dinhdatvn96@gmail.com (D.D.V.); suminp0309@gmail.com (S.P.); jungoh@pknu.ac.kr (J.O.); 4 Smart Gym-Based Translational Research Center for Active Senior’s Healthcare, Pukyong National University, Busan 48513, Republic of Korea; jaeyeopchoi@pknu.ac.kr (J.C.); tanhung0506@gmail.com (T.H.V.); 5 School of Mechanical Engineering, Hanoi University of Science and Technology, Hanoi 100000, Vietnam; son.truonghoanh@hust.edu.vn; 6 Ohlabs Corporation, Busan 48513, Republic of Korea

**Keywords:** waterstream, automotive SAM system, non-immersive transducer probe, SAM in-line inspection, ultrasonic non-destructive testing

## Abstract

A scanning acoustic microscopy (SAM) system is a common non-destructive instrument which is used to evaluate the material quality in scientific and industrial applications. Technically, the tested sample is immersed in water during the scanning process. Therefore, a robot arm is incorporated into the SAM system to transfer the sample for in-line inspection, which makes the system complex and increases time consumption. The main aim of this study is to develop a novel water probe for the SAM system, that is, a waterstream. During the scanning process, water was supplied using a waterstream instead of immersing the sample in the water, which leads to a simple design of an automotive SAM system and a reduction in time consumption. In addition, using a waterstream in the SAM system can avoid contamination of the sample due to immersion in water for long-time scanning. Waterstream was designed based on the measured focal length calculation of the transducer and simulated to investigate the internal flow characteristics. To validate the simulation results, the waterstream was prototyped and applied to the TSAM-400 and W-FSAM traditional and fast SAM systems to successfully image some samples such as carbon fiber-reinforced polymers, a printed circuit board, and a 6-inch wafer. These results demonstrate the design method of the water probe applied to the SAM system.

## 1. Introduction

Scanning acoustic microscopy (SAM) is a powerful, non-destructive instrument for material quality evaluation owing to its capabilities of visualizing the internal structure of a sample without destruction. In the 1970s, the SAM system was developed by Lemons and Quate [1]; it was used for imaging inside solid samples and biological tissues [2,3]. Nowadays, SAM is a well-accepted imaging instrument for scientific and industrial applications.

In scientific applications, several studies have utilized the SAM system to analyze the biological characteristics and quality of samples. The SAM system has been employed to characterize both soft tissues [4,5] and hard tissues [6,7]. Kundu et al. [8] and Soon et al. [9] used the SAM system to visualize live cells and determine their mechanical properties. The conditions of biological matter were illustrated using the SAM system [10,11]. Moreover, the SAM system was used to measure cell properties, such as sound speed, thickness, and density [12]. Using the C-scan image of the SAM system, the structural homogeneity of a sample was examined by identifying the shape and size of internal defects. The delamination between mold compound and lead frame of an integrated circuit (IC) chip was visualized in a C-scan image [13,14,15]. Wang et al. [16] used the SAM system to evaluate the quality of the flip chip assembly process. By interpreting the C-scan image, the delamination region was detected, thereby evaluating the quality of a printed circuit board (PCB). Owing to the complicated manufacturing process of wafers, internal defects can occur, which can be observed using the SAM system [17]. Twerdowski et al. [18] used the SAM system to locate the disbonded and weakly bonded regions inside the wafer. Noh et al. [19] evaluated the bonding quality by using the SAM system to visualize bonding behavior. In addition, the SAM system was proposed for the investigation of microstructure damage [20] and evaluation of composite adhesive joints [21] in a carbon fiber-reinforced polymer (CFRP) plate. By using the SAM system, the quality of other samples was evaluated such as welded joints [22,23,24,25,26], coating materials [27], and batteries [28].

For industrial applications, many commercial SAM systems have been developed, such as those by KSI, Honda, Sonix, Nordson, and PVA TePla. Their systems have been used for product inspection in mass production. The automotive SAM system was proposed to enhance the system’s capabilities in in-line inspection. In this way, a robot arm is incorporated into the SAM system to transfer the sample, for instance, the SAM 300 system (PVA TePla). The wafer sample was transferred from the tray to the fixture. To implement the scanning process, the fixture was controlled to fully immerse the sample into the water. By integrating a robot arm and fixture into the SAM system, the entire system becomes complicated, thereby increasing time consumption.

Technically, water is used as an environment for ultrasound wave propagation from transducer to sample. Therefore, the sample is fully immersed and the transducer is semi-immersed in water in most SAM systems during the scanning process. The sample being fully immersed in water could lead to damage due to contamination such as oxidation, especially for metal material. To overcome the aforementioned problems, a water probe has been proposed for use in the SAM system. However, water probes are only used in commercial SAM systems (Sonoscan, Nordson) and there is no research focused on water probe development for SAM systems. According to the commercial version, the water pressure at the outlet of the water probe is high, which may cause damage to the sample’s top surface, especially for soft materials.

This study aims to design and fabricate a novel water probe to be used for the SAM system, that is, a waterstream. The waterstream supplies a continuous flow between the transducer and sample, which maintain acoustic coupling during the scanning process. In other words, the tested sample is not immersed in water, which can avoid damage of the sample due to contamination. In addition, using a waterstream leads to a simple design of the automotive SAM system and reduces time consumption by transferring the sample by a standard mechanism (i.e., conveyor). The waterstream was designed based on the water domain that was simulated to investigate the internal flow characteristics. The water domain was modeled using the measured focal length calculation of the transducer. Using the PISO (Pressure Implicit of Splitting Operator) algorithm integrated into the open-source OpenFOAM 8 software, the instantaneous values of velocity and pressure were plotted. Furthermore, the water pressure at the outlet (sample top surface) was determined to have a value approximately equal to atmospheric pressure, which could protect the sample’s top surface, particularly for soft materials (i.e., soft tissues). Based on the simulation results of the water domain, the waterstream was prototyped and applied to the traditional SAM (TSAM−400) and wafer fast SAM (W−FSAM) systems (Ohlabs Corp., Busan, Republic of Korea) to conduct experiments. The setup of the water supply system was simple; it comprised an immersion pump with a manual valve. Using the SAM system with waterstream, CFRP, PCB, and 6-inch wafer samples were successfully captured.

The remainder of the paper is organized as follows. Section 2 describes the schematic of the SAM system and focal length calculation of the transducer. Based on the results of Section 2, the water domain is modeled and set up for simulation in Section 3. Section 4 shows the simulation results and waterstream prototype. The waterstream is used for two SAM systems to conduct the experiments that are presented in Section 5. Finally, the conclusions are expressed in Section 6.

## 2. Schematic of Scanning Acoustic Microscopy (SAM) System and Focal Length Calculation

### 2.1. Scanning Acoustic Microscopy (SAM) System

The operation of the SAM system is built based on the ultrasound (US) transducer characteristics that uses the sensitivity of US waves for visualizing the internal structure of the sample without deconstruction. The US transducer propagates US waves to the sample through the water and receives the echo signals reflected off the sample, which are converted to digital signals. The transducer is attached to the scanning module and the sample is fixed to the sample table. The scanning module can be adjusted along the *z*-axis to define the focal zone. Technically, the scanning module consists of two linear motions along the *x*- and *y*-axes. During the scanning process, the scanning module is controlled in a sequence to generate the scanning images. When the scanning module completes one line along the *x*-axis, it will move one step along the *y*-axis.

**Figure 1:**
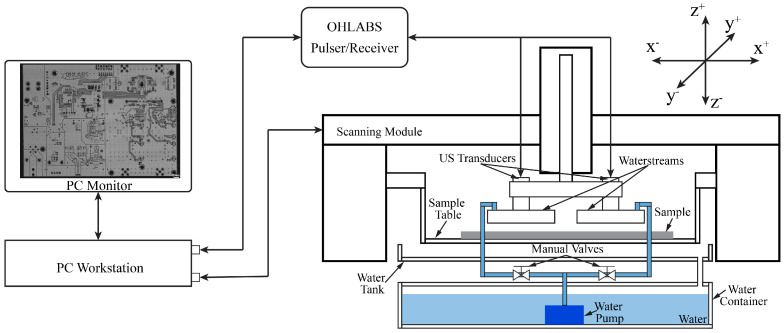
Schematic of the TSAM−400 system with waterstream.

**Figure 2:**
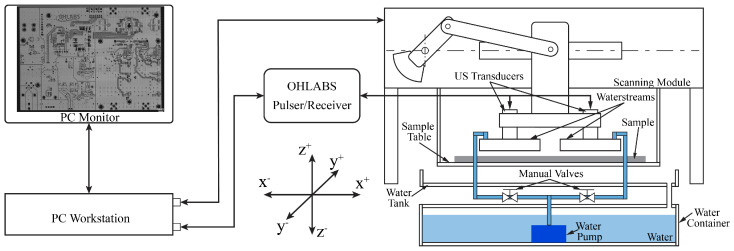
Schematic of the W−FSAM system with waterstream.

In previous studies, traditional SAM (TSAM−400) and fast SAM (W−FSAM) systems were developed to successfully capture samples that were fully immersed in water. The motion along the *y*-axis of both systems was implemented by a ball-screw mechanism. TSAM−400 uses a linear motor to develop linear motion along the *x*-axis, which evaluates the quality of spot-welded sheets [29]. To enhance the efficiency of the SAM system with regard to in-line inspection, the W−FSAM system was designed by exploiting the slider-crank mechanism to conduct high-speed motion along the *x*-axis. Using the W−FSAM system, the scanning time was significantly reduced while maintaining the high resolution of the image results. In this study, a waterstream is used to supply water instead of immersing the sample in water during the scanning process; it is applied to the TSAM−400 and W−FSAM systems. Figure 1 and Figure 2 show schematics of the TSAM−400 and W−FSAM systems using the waterstream, respectively. The waterstream is attached to the transducer. Using an immersion pump, water is supplied to the waterstream from a water container. The flow input is controlled using a manual valve to maintain the flow velocity at the inlet. Water returns to the water container via a water tank, as shown in Figure 1 and Figure 2.

### 2.2. Focal Length Calculation of the Transducer

**Figure 3:**
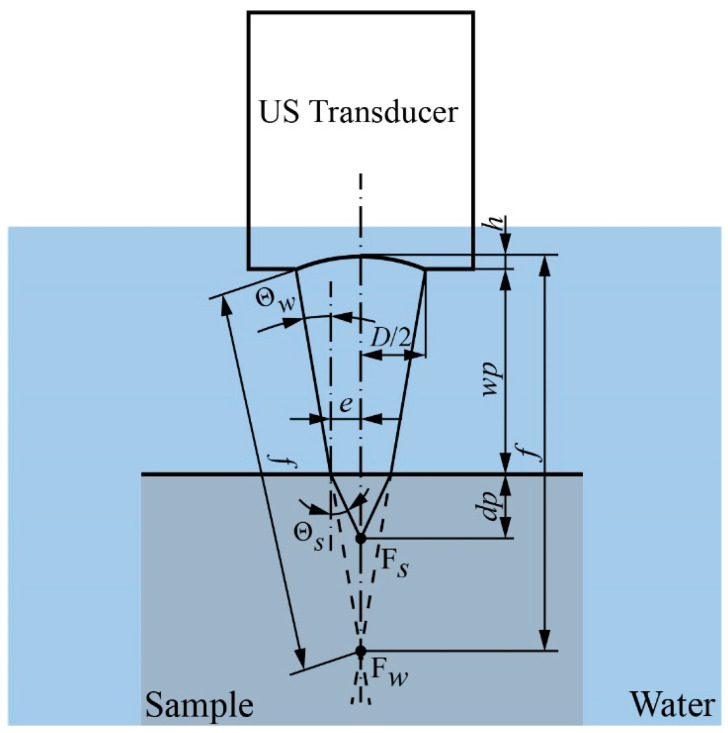
The measured focal length of transducer inside sample.

To generate high-contrast images, the transducer and sample are aligned along the *z*-axis to define the focal zone before the scanning process. According to the transducer technical report, the focal length (*f*) measured in water is given. For a specific sample, the measured focal length of the transducer depends on the material’s characteristics. The acoustic wave propagates to the top surface of the sample at an incident angle (aperture angle, Θ*_w_*) through the water. Due to the difference in the acoustic properties of the water and sample material, the acoustic wave is refracted when it goes inside the sample. The refracted angle (Θ*_s_*) is calculated using Snell’s law [30,31], as defined by

(1) $$\frac{\sin\left(\Theta_{w}\right)}{c_{w}}=\frac{\sin\left(\Theta_{s}\right)}{c_{s}}$$

where *c_w_*, *c_s_* denote the longitudinal velocities of acoustic waves propagating in the water and sample, respectively. As the acoustic velocity in most materials is higher than that in water, the focal length in the sample is effectively shortened. Figure 3 depicts the refraction at the boundary of the water and sample (the sample top surface) of the acoustic wave generated by the US transducer.

The measured focal length inside the sample (*f_s_*) is determined as follows:

(2) $$f_{s}=h+wp+dp$$

where *h* and *dp* are calculated as

(3) $$h=f\left(1-\cos\left(\Theta_{w}\right)\right)$$

(4) $$dp=\frac{e}{\tan{(\Theta }_{s})}$$

(5) $$e=\frac{D(f-h-wp)}{2(f-h)}$$

where *D* and *wp* stand for element size of the transducer and distance from the transducer to the sample (top surface), respectively. The waterstream is designed based on the *wp* value, that is defined by

(6) $$wp=f\cos\Theta_{w}\left(1-\frac{2dp(\tan\Theta_{s})}{D}\right)$$

(7) $$\Theta_{w}=\sin^{-1}\left(\frac{D}{2f}\right)$$

(8) $$\Theta_{s}=\sin^{-1}\left(\frac{Dc_{s}}{2fc_{w}}\right)$$

## 3. Modeling and Simulation Setup of Waterstream

### 3.1. Water Domain Modeling

The waterstream is used to continuously maintain the water environment between the transducer and sample during the scanning process. Water is supplied at the inlet through one-touch fitting, whereas the outlet is defined at the top surface of the sample. The water domain is modeled based on the *wp* value that depends on the transducer and sample characteristics. In this study, a 100 MHz focused transducer with focal length (*f*) of 8 mm and element size (*D*) of 3 mm was used, thereby generating an aperture angle (Θ*_w_*) of 10.81°. To increase the ability of the waterstream, the water domain was modeled with the minimum value of *wp*, corresponding to the maximum values of *dp* and *c_s_*. The maximum value of *dp* was considered as the penetration depth of the transducer; the value was 0.4 mm, considering the 100 MHz focused transducer [32]. The value of *c_s_* was 4660 m/s, with regard to the copper material. All the experiments were conducted at room temperature (25 °C); thus, the value of *c_w_* was set to 1500 m/s. Therefore, the value of *wp* was 6.4 mm.

**Figure 4:**
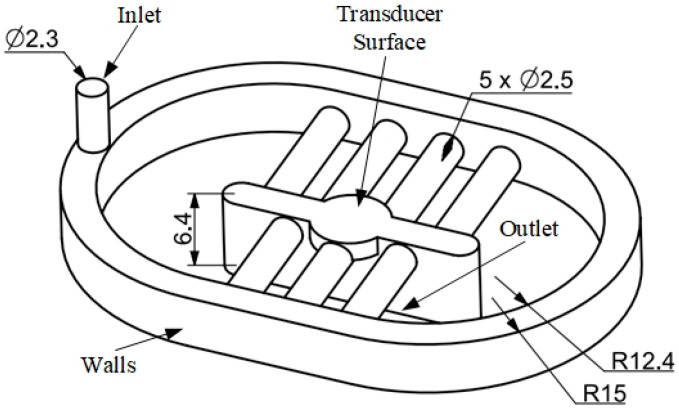
Water domain modeling.

Figure 4 shows the modeling of the water domain. The inlet diameter was 2.3 mm corresponding to the hole diameter obtained through one-touch fitting. The distance between the transducer surface and outlet was set equal to *wp*. Water was flowed from the inlet to the outlet through the wall boundary that was modeled to cover the transducer outside diameter of 22.9 mm.

### 3.2. Governing Equations

In this study, all the experiments are conducted at room temperature (25 °C); thus, the water flow is incompressible. To determine velocity and pressure, the continuity and momentum equations are established based on the Navier–Stokes equations, that are expressed by

(9) $$\frac{\partial u}{\partial x}+\frac{\partial v}{\partial y}+\frac{\partial w}{\partial z}=0$$

(10) $$\frac{\partial u}{\partial t}+\frac{\partial (uu)}{\partial x}+\frac{\partial (vu)}{\partial y}+\frac{\partial (wu)}{\partial z}=g_{x}-\frac{\partial p}{\partial x}+v\left(\frac{\partial^{2}u}{\partial x^{2}}+\frac{\partial^{2}u}{\partial y^{2}}+\frac{\partial^{2}u}{\partial z^{2}}\right)$$

(11) $$\frac{\partial v}{\partial t}+\frac{\partial (uv)}{\partial x}+\frac{\partial (vv)}{\partial y}+\frac{\partial (wv)}{\partial z}=g_{y}-\frac{\partial p}{\partial y}+v\left(\frac{\partial^{2}v}{\partial x^{2}}+\frac{\partial^{2}v}{\partial y^{2}}+\frac{\partial^{2}v}{\partial z^{2}}\right)$$

(12) $$\frac{\partial w}{\partial t}+\frac{\partial (uw)}{\partial x}+\frac{\partial (vw)}{\partial y}+\frac{\partial (ww)}{\partial z}=g_{z}-\frac{\partial p}{\partial z}+v\left(\frac{\partial^{2}w}{\partial x^{2}}+\frac{\partial^{2}w}{\partial y^{2}}+\frac{\partial^{2}w}{\partial z^{2}}\right)$$

where *u*, *v*, and *w* denote the flow velocities along the *x*-, *y*-, and *z*-axes, respectively. The gravitation acceleration along the *j*-axis (*j* = x, y, and z) is given by *g_j_*. *p* and υ denote the kinematic pressure and kinematic viscosity, respectively. The Reynolds number (*Re*) is used to predict flow patterns, which is calculated by

(13) $$Re=\frac{Ud}{v}$$

where *d* and *U* are the inlet diameter and magnitude of the flow velocity, respectively. In this study, the flow velocity was maintained at 12 m/s using a manual valve. The water kinematic viscosity (υ) was 10^−6^ m^2^/s. Therefore, *Re* was 27,600, which represents turbulent flow. The kappa-epsilon (*k-ε*) model is widely used to describe turbulent flow with high Reynolds numbers. Two transport equations of turbulent kinetic energy (*k*) and dissipation (*ε*) are expressed to represent turbulent properties. To conduct the numerical simulation of the *k-ε* model, the initial conditions are defined as follows.

(14) $$k=\frac{3}{2}{\left(UI\right)}^{2}$$

where *I* denotes the initial turbulent intensity, given by

(15) $$I=0.16{Re}^{-\frac{1}{8}}$$

(16) $$\varepsilon =\frac{c_{\mu }^{\frac{3}{4}}k^{\frac{3}{2}}}{l}$$

where *c_μ_* is a *k-ε* model parameter with a value of 0.09, and *l* is the turbulent length scale, which is calculated as

(17) l=0.07d

### 3.3. Simulation Setup

**Figure 5:**
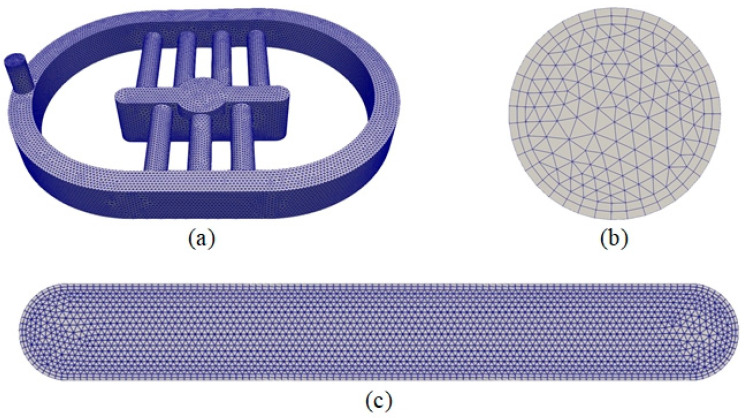
Mesh construction of (**a**) water domain, (**b**) inlet, and (**c**) outlet.

Before the simulation process, the water domain was created and mesh constructions were generated by using the open-source SALOME software version 9.7.0. Figure 5a shows the mesh construction of the water domain that contains 295,161 elements. The mesh constructions of the inlet and outlet are shown in Figure 5b and Figure 5c, respectively. The convergence and runtime depend on the element sizes, that were set with minimum and maximum values of 0.01 and 0.07 mm, respectively.

The *k-ε* turbulent model was simulated using OpenFOAM. The pressure and velocity values were achieved through the PISO scheme. The initial pressure was set to 101.325 m^2^/s^2^ (atmospheric pressure in kinematic pressure). The velocities on the wall boundaries were defined as no-slip condition. The initial conditions of the simulation are listed in Table 1.

## 4. Simulation Results and Waterstream Prototype

### 4.1. Simulation Results

**Figure 6:**
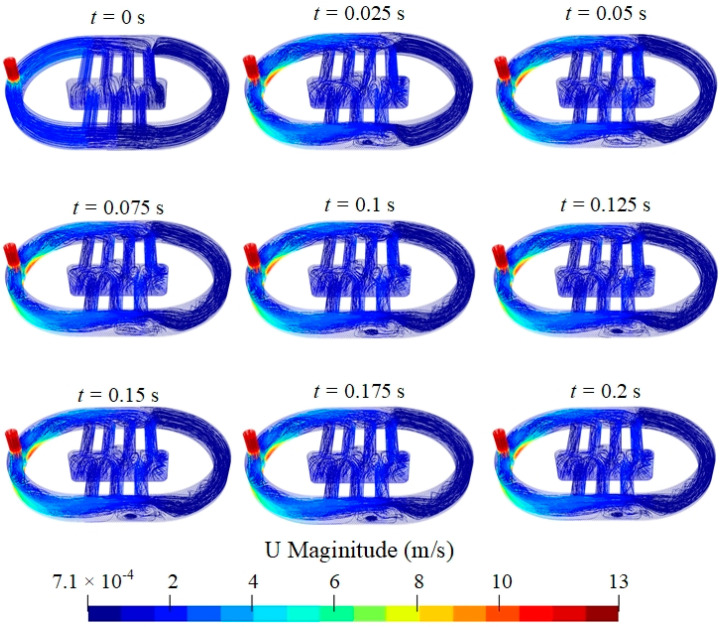
Velocity distribution inside water domain plotted as streamlines.

In this study, a waterstream is used to maintain the water environment between the transducer and sample during the scanning process. To investigate the flow characteristics, the instantaneous values of velocity and pressure in the water domain were determined and saved at each time step. Using OpenFOAM, these values were plotted at *t* = 250 × *n* × Δ*t*, where *n* denotes integers from 0 to 8. Figure 6 shows the instantaneous velocity distribution inside the water domain, which is plotted as a streamline. At *t* = 0 s, water is injected from the inlet at the highest velocity. Owing to the high flow velocity, turbulence was generated inside the water domain. When the flow moved toward the outlet, the turbulence was dissipated because of the flow velocity decrease. From *t* = 0.1 s, the flow pattern was stable, thus forming a continuous flow that moved to the outlet.

**Figure 7:**
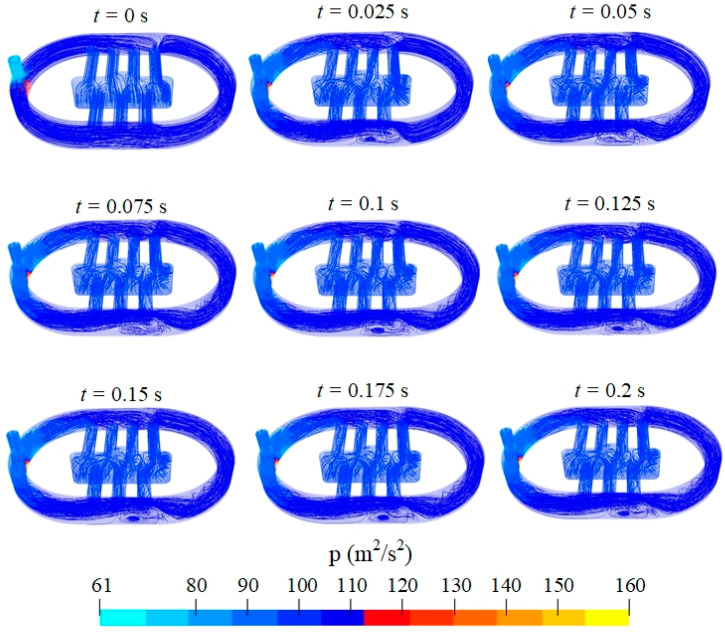
Pressure distribution inside water domain plotted as streamlines.

The pressure distribution inside the water domain is shown in Figure 7. According to Bernoulli’s principle, low-pressure regions were obtained at the areas that exhibited high flow velocity. In other words, low pressure appeared in turbulent areas. The flow velocity in the inlet throat was maintained at a high value for all the time steps, thereby generating low pressure in the inlet throat. Similar to the velocity distribution, a stable state of pressure was obtained from *t* = 0.1 s. An animation video of the velocity and pressure distributions was recorded for illustration (Supplementary Movie S1).

**Figure 8:**
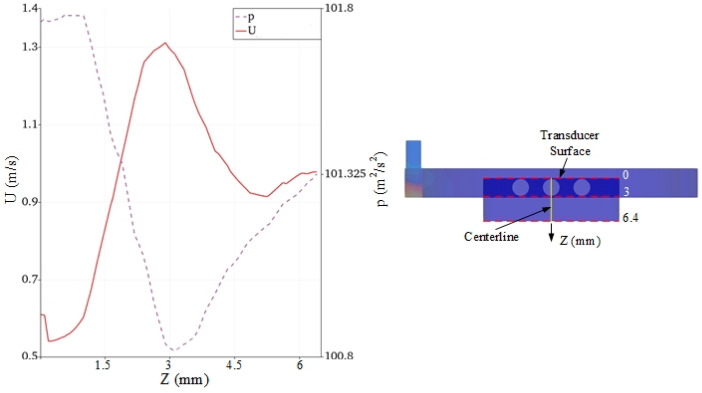
The velocity and pressure distribution along the centerline between the transducer and outlet.

For further understanding the velocity and pressure behaviors inside the water domain, the velocity and pressure profiles along the centerline between the transducer surface and outlet are shown in Figure 8. The velocity was the highest at Z = 3 mm, near the middle orifice. The flow velocity decreased to 0.55 m/s when the water level reached to transducer surface. As expected, the pressure behavior plotted in contrast to the velocity profile; this indicates that the pressure increased when the velocity decreased and vice versa. At the outlet, the pressure was approximately equal to atmospheric pressure, whereas the flow was continuous at 1 m/s.

According to the simulation results, the flow was stable after 0.1 s, which indicated the scanning process could be started immediately without delay of the water supply. The flow behavior demonstrated that the acoustic propagation was continuous between transducer and sample, which resulted in no missing signal in the data collection for image processing. In addition, the pressure at the outlet was approximately 1 atm, potentially protecting the sample’s top surface from damage.

### 4.2. Waterstream Prototype

**Figure 9:**
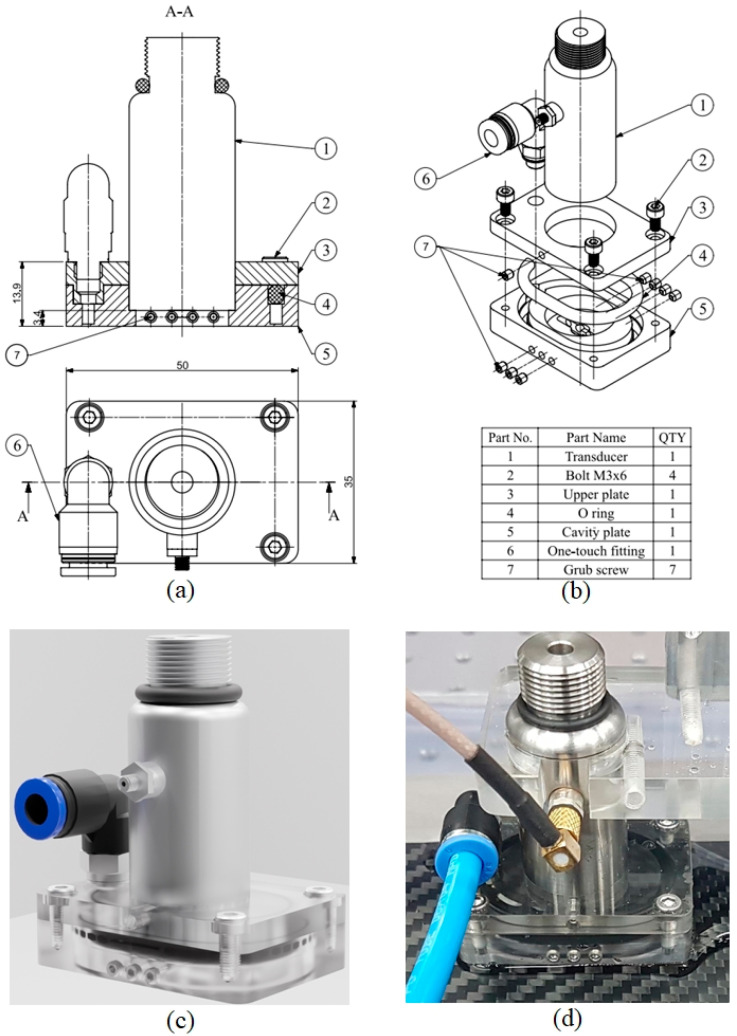
Waterstream design and prototype: (**a**) 2D drawing, (**b**) 3D exploded drawing, (**c**) rendered concept, (**d**) prototype.

The waterstream is designed based on the modeling parameters, which cover the entire water domain. To simplify the design and fabrication processes, the concept of the waterstream includes seven components, as shown in Figure 9a,b. One-touch fitting was fixed on the upper plate by a thread joint, which defined the inlet of the water domain. The walls and outlet were created in the cavity plate. Figure 9a shows a two-dimensional (2D) drawing of the waterstream. The upper and cavity plates were connected by bolts. The waterstream was mounted on the transducer using a grub screw. Figure 9b shows an exploded view of a three-dimensional (3D) drawing of the waterstream. An O-ring was used between the upper and cavity plates to restrict water overflow. The grub screws were fixed on both sides of the cavity plate to curb the water leakage problem.

During the scanning process, the transducer was controlled to move in a sequence. To facilitate the motions, the gap between the bottom face of the cavity plate and top face of the sample was set at a minimum value of 3 mm, and thus, the distance between the transducer surface and bottom face of the cavity plate was 3.4 mm, as shown in Figure 9a. Figure 9c shows the rendered concept of a waterstream. The upper and cavity plates were fabricated using acrylic materials. Finally, the waterstream was assembled as shown in Figure 9d.

## 5. Experimental Results

The TSAM-400 and W-FSAM systems were used with the waterstream to validate the simulation results. The water was supplied to two waterstreams using the immersion pump. Two independent manual valves were used to maintain the flow velocity through one-touch fitting. To demonstrate the capabilities of the waterstream, some samples were successfully captured using the SAM systems: a CFRP, PCB, and 6-inch wafer.

### 5.1. Waterstream for the TSAM−400 System

**Figure 10:**
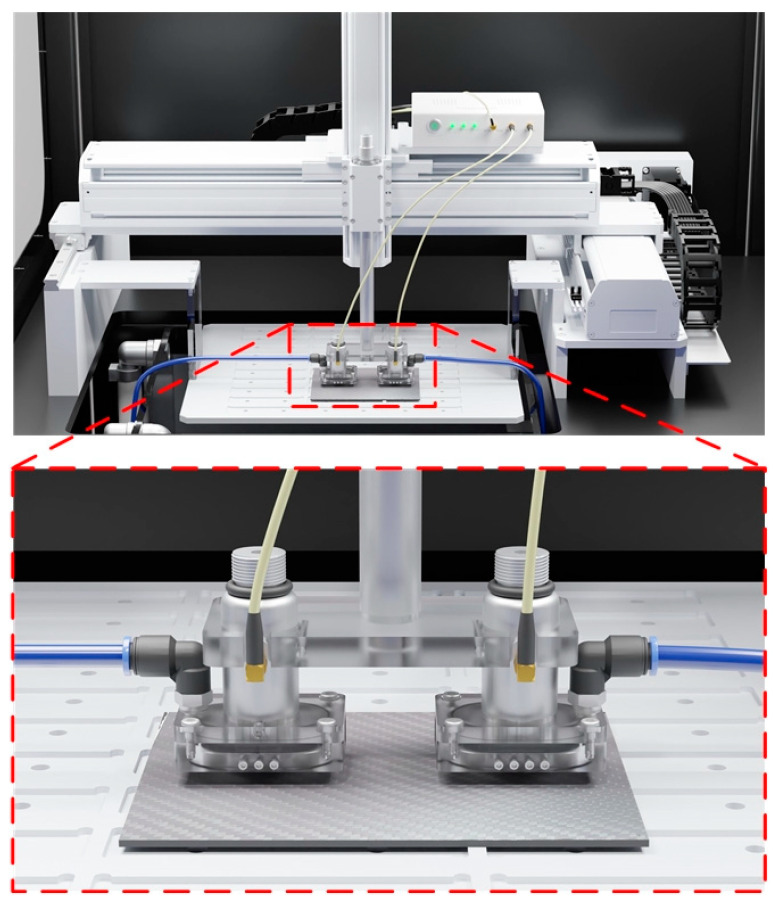
Rendered image of TSAM−400 system with two waterstreams.

**Figure 11:**
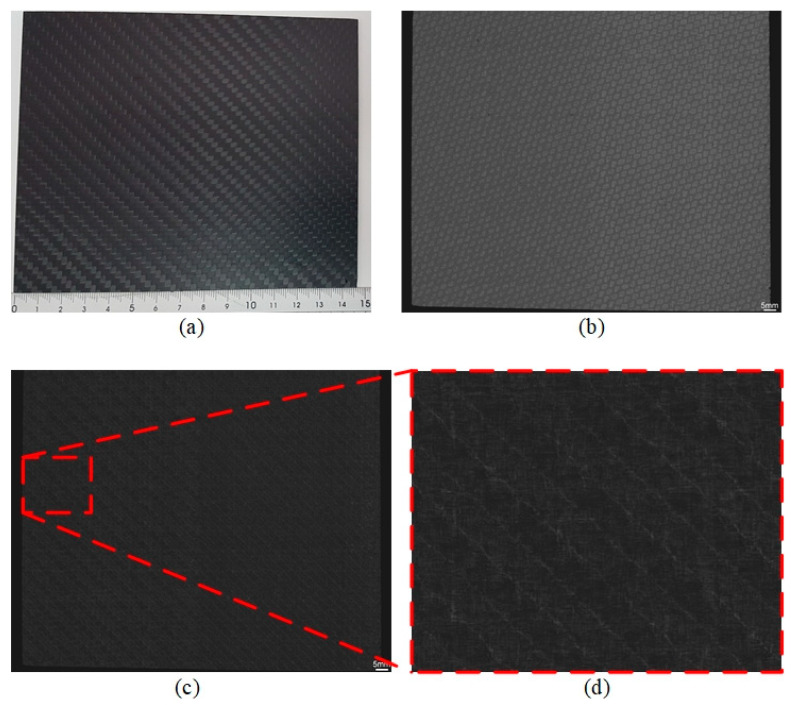
(**a**) CFRP sample: C-scan images of (**b**) top surface, (**c**) underlayer, and (**d**) enlarged view of underlayer.

A CFRP sample was prepared to be scanned using the TSAM−400 system with waterstream. To reduce the scanning time, two identical transducers were arranged within a distance of 77.5 mm along the *x*-axis, as shown in Figure 10. Figure 11a shows the CFRP sample with an area of 146 × 122 mm^2^. To entirely cover the sample along the *x*-axis, the linear motor was implemented to travel 77.5 mm at 4.5 Hz, which were the same values used in a previous study [29].

Figure 11b,c show the scanning images of the top surface and underlayer of the CRFP sample, respectively. These results indicate the good adhesive quality between the top surface and underlayer (no delamination), as shown in Figure 11c. In addition, the fiber orientation is illustrated in the underlayer image, as shown in Figure 11d.

### 5.2. Waterstream for the W−FSAM System

The W−FSAM system was developed for providing acceptable scanning images in a short time. Owing to the fast movement of the transducer, the scanning time was significantly reduced. To demonstrate the capabilities of the waterstream applied to the W−FSAM system, the CFRP sample was scanned with the same scanning parameters as those of the TSAM−400 system. Using the W−FSAM system, the image was successfully captured within approximately 6.4 min (Supplementary Movie S2), thereby reducing the scanning time by 50% compared to the TSAM−400 system.

**Figure 12:**
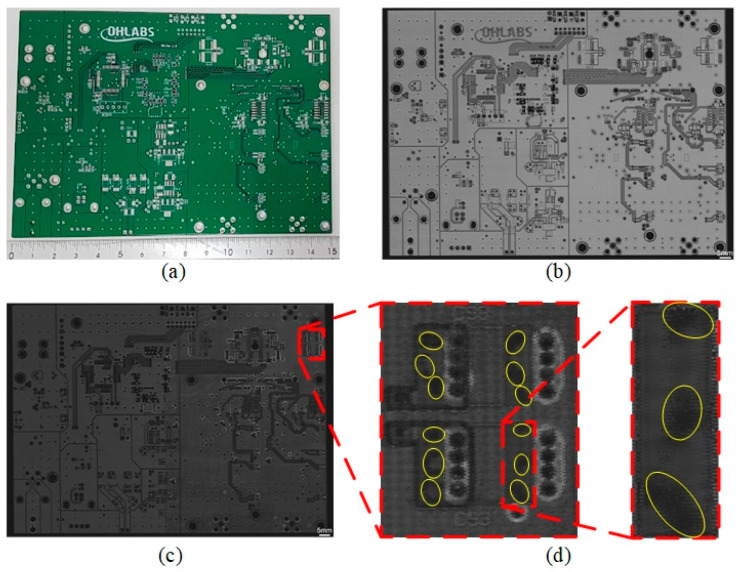
(**a**) PCB sample, (**b**) top surface C-scan image, (**c**) underlayer C-scan image, and (**d**) enlarged view of soldering area.

The quality of the PCB sample was evaluated using the W−FSAM system with waterstream. Figure 12a shows the PCB sample with an area of 150 × 109 mm^2^. The accuracy of the component positions is illustrated in the C-scan image of the top surface, as shown in Figure 12b. Figure 12c shows the underlayer C-scan image, which can be used to evaluate the soldering quality of the PCB. Delamination regions were detected in the soldering area, as highlighted by the yellow ellipses in Figure 12d. The yellow ellipses were darker than those in remained regions, which indicated that the amplitude of reflected signal at yellow ellipses was stronger. That means the structure at these regions was nonhomogeneous. In other words, these regions were the delamination. Compared to the results of immersing the PCB in water using the FSAM system [33], these images were identical, and were obtained at the same speed. Using the W−FSAM system, only one scanning process was conducted to successfully evaluate the quality of the entire PCB, which enabled in-line inspection in PCB mass production.

**Figure 13:**
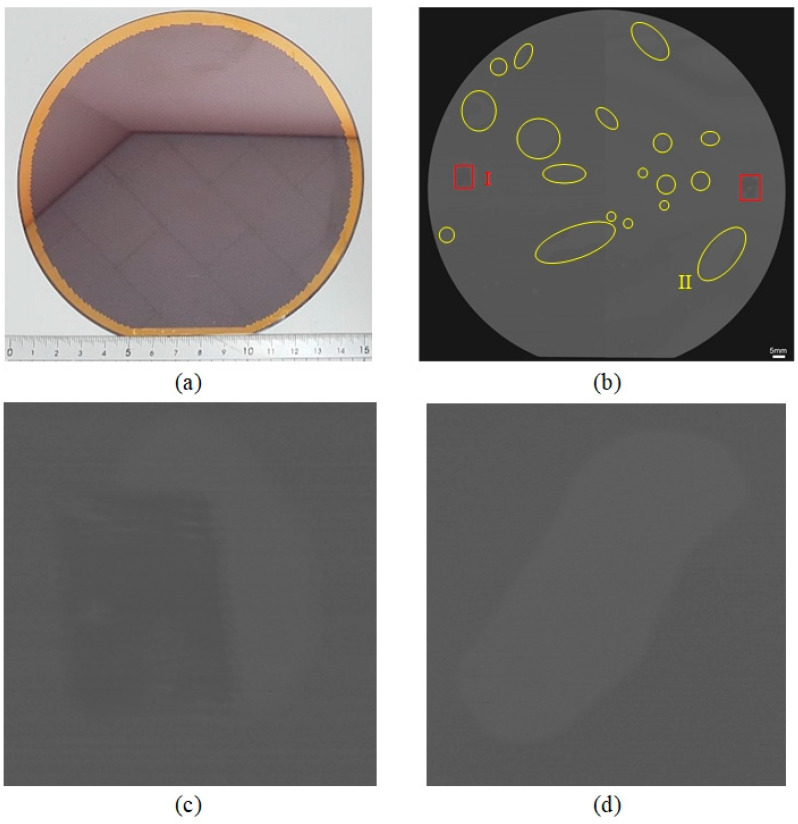
(**a**) The 6-inch wafer sample, (**b**) C-scan image of wafer, (**c**) enlarged view of I area, (**d**) enlarged view of II area.

The 6-inch wafer sample was prepared to be scanned using the W−FSAM system, as shown in Figure 13a. The scanning parameters were set at 155 × 146 mm^2^, 0.04 mm, and 8 Hz, corresponding to the scanning area, step size, and B-scan frame rate, respectively. The scanning time was approximately 7.6 min (Supplementary Movie S3). Figure 13b shows the C-scan image of the wafer, which is interpreted to evaluate the quality of the wafer. There were two defects inside the wafer sample, as highlighted by the red rectangles. Some delamination areas were detected in the C-scan image, as highlighted by the yellow ellipses. Figure 13c,d show an enlarged view of the defect (I) and delamination (II) areas, respectively. These results indicate that the sample quality is unacceptable.

## 6. Conclusions

In this study, a novel water probe (waterstream) was designed and fabricated for a high-frequency focused transducer, which was used for the SAM system to enhance its capabilities in in-line inspection. The waterstream was developed based on the water domain that was modeled by the measured focal length calculation of the transducer. A numerical solution was conducted to investigate the internal flow characteristics inside the water domain. The simulation was performed based on the PISO algorithm using OpenFOAM. The simulation results indicated that continuous flow was maintained between the transducer surface and the outlet. The flow velocity at the outlet was approximately 1 m/s while the pressure was equal to atmospheric pressure.

The simulation results were validated by experiments. Based on the water domain model, the waterstream was designed, fabricated, and applied to the traditional and fast SAM systems. Some samples were successfully imaged using the TSAM−400 and W−FSAM systems with waterstream, which were identical to those in the case of immersing the sample in water. The results demonstrate the simulation results and indicate that these systems can be used for in-line inspection. Normally, when the SAM system was used to scan a sample immersed in water, a robot arm was implemented for transferring the sample, which created a complicated system and increased time consumption. Using a waterstream in the SAM system, a conveyor was used to transfer the sample to conduct an automotive inspection, which led to a simple system and reduced time consumption. In addition, the sample was scanned without immersing it in water, which could protect the sample from contaminants. Furthermore, the water pressure at the top surface of the sample was approximately equal to atmospheric pressure, which could help avoid damage to the sample, especially for soft samples. These results demonstrate the potential application of waterstreams in automotive SAM systems.

In order to extend the research based on the simulation and experimental results, future studies are suggested. First, a waterstream can be developed for transducers based on their measured focal length and internal flow simulation. Second, the SAM system with waterstream can be incorporated into an industrial robot for imaging samples with surface curvature.

## Figures and Tables

**Table 1:** The initial conditions of simulation.

Input	Value
Simulation time (*t*)	0.2 s
Time step (Δ*t*)	10^−6^ s
Flow velocity (*U*)	12 m/s
Outlet pressure (*p*)	101.325 m^2^/s^2^
Kinetic energy (*k*)	0.429 m^2^/s^2^
Dissipation rate (*ε*)	286.785 m^2^/s^3^

## Data Availability

Dataset available on request from the authors.

## Supplementary Materials

The following supporting information can be downloaded at https://doi.org/10.5281/zenodo.11272954.
